# Divine intervention? A Cochrane review on intercessory prayer gone beyond science and reason

**DOI:** 10.1186/1477-5751-8-7

**Published:** 2009-06-10

**Authors:** Karsten Juhl Jørgensen, Asbjørn Hróbjartsson, Peter C Gøtzsche

**Affiliations:** 1 The Nordic Cochrane Centre, Rigshospitalet, Dept. 3343, Blegdamsvej 9, DK-2100 Copenhagen, Denmark

## Abstract

We discuss in this commentary a recent Cochrane review of 10 randomised trials aimed at testing the religious belief that praying to a god can help those who are prayed for. The review concluded that the available studies merit additional research. However, the review presented a scientifically unsound mixture of theological and scientific arguments, and two of the included trials that had a large impact on the findings had problems that were not described in the review. The review fails to live up to the high standards required for Cochrane reviews.

## Commentary

The religious belief that praying to a god can help those who are prayed for may be studied scientifically in randomised trials, but it raises important problems. One of the problems is that researchers who investigate interventions that have no credible mechanism need to interpret positive results very carefully. A statistically significant result is less convincing in a trial of prayer or homoeopathy than in a trial of a new non-steroidal, anti-inflammatory drug that has a similar molecular structure as existing drugs with a documented effect and that has been effective in animal studies [1].

This important line of reasoning is formalized in Bayesian statistics that operate with an *a priori *likelihood that is modified according to the *a posteriori *P value that the trial generates. From a scientific perspective, the *a priori *likelihood that prayer could be effective is extremely small, as it involves three assumptions that are all unlikely to be true. First, the existence of a god; second, that prayer can somehow travel in space and reach this god, or that it works through another mechanism unknown to science; third, that this god is responsive to prayer and can influence at a distance what would otherwise have happened. Most researchers would find it futile to perform randomised trials of the effect of prayer on those prayed for. Any observed effect would more likely be due to the play of chance, bias or fraud, than to divine intervention. It would be more fruitful to study possible psychologically soothing effects among the prayers themselves. In any case, the *modus operandi *of trials of prayer must be to perform the evaluation from a scientific position without interference of theological arguments or reservations.

A Cochrane review of ten randomised trials (7646 patients) of intercessory prayer concludes that the evidence justifies further trials [2]. However, the authors mix theological arguments with scientific reasoning, are logically inconsistent and do not relate crucial information about the included trials to the reader. The authors have apparently not discovered that the largest "trial" was meant to amuse rather than being scientific evidence, and that a suspicion of fraud has been raised against another large trial included in the review. Finally, if we were to accept the authors' theological reasoning, all scientific research would become meaningless, and we therefore examine their main arguments below.

### Methodology of the Cochrane review

The authors state in the background section that "outcomes of trials of prayer cannot be interpreted as 'proof/disproof' of God's response to those praying", and that what they attempt to quantify is an "effect of prayer not dependent on divine intervention" [2]. It is difficult to understand what they mean by this. Why would people pray to a god if an effect of prayer is not caused by divine intervention, and what would then be the causal mechanism? The authors provide no explanation, and it is hard to imagine how prayer for ill people located at the other side of the globe [2], and who were unaware that someone prayed for them, could have an effect without assuming divine intervention. It is also hard to accept that a god would help Peter in bed A because someone randomly decided to pray for him, but not the less fortunate Paul in bed B. If we were made in a god's image, as some religions claim, one would expect us to share ethical values, and such an action would conflict with most people's sense of fairness.

The authors contradict themselves when they say that their review focuses on people "setting time aside to communicate with God", as the review is not about divine intervention. They are also inconsistent when they note that "If understanding of God is as limited as the Holy Literature suggests (1 Corinthians 13:12), the consequences of divine intervention may be considerably more subtle than could be measured in the crude results of a trial" and that "It could be that any effect of prayer are due to elements beyond present scientific understanding". If these were real concerns, the authors should not have undertaken the review, as the reservations mean that people who do trials of prayer cannot rely on what they observe. Such arguments are also used by practitioners of alternative medicine, and in the theory of science, this approach is called immunization of the research hypothesis. It means that regardless of which experimental results are obtained, believers will be unaffected and will continue to claim, with the same strength as before, that their intervention is effective.

Another statement is also mystifying. The authors write that "An omnipotent God would make concealment of allocation impossible and may be noncompliant with the limitations of a randomised trial (Psalm 106:14,15, Job 42:2)". As such a god could interfere with the experimental set-up, it is difficult to understand why the authors excluded trials in which the treatment allocation was not concealed, and why they bothered to discuss the level of concealment in the trials they included.

### Included trials

We have not checked all ten included trials but noted that the largest one was published in BMJ's Christmas issue [3]. This trial seems to be meant to amuse rather than being a scientific study [4], in line with the tradition of this special issue, as the trial evaluated the effect of prayer taking place 4–10 years *after *the patients had either left the hospital alive, or had died from their bloodstream infection. Thus, the trial evaluated the effect of *retroactive *intercessory prayer using historical data and its author argued that we cannot assume "that God is limited by a linear time" [3]. The authors of the Cochrane review did not mention anywhere in their review that the patients were randomised many years after their outcomes had occurred and did not discuss the likelihood that time can go backwards and that prayer can wake the dead.

The author of the retrospective study noted subsequently that "if the pre-trial probability is infinitesimally low, the results of the trial will not really change it, and the trial should not be performed. This, to my mind, turns the article into a non-study" [4]. We agree. The non-study "found" a non-significant reduction in death for those prayed for (relative risk 0.93, 95% confidence interval 0.84 to 1.03) [3], but it carried 75% of the weight in the meta-analysis of this outcome in the Cochrane review, leading to a statistically significant effect [2].

In a subsequent Christmas issue, authors with an interest in alternative medicine, prayer and healing tried to explain why the results of the retroactive study could be true, using arguments from quantum theory [5]. They seemed to take their own arguments seriously but they are entirely unreasonable and demonstrate a poor understanding of quantum theory [6]. Down-to-earth, it should not be too difficult to realise that prayer cannot make dead patients come to life again. In fact, all the randomisation did was to divide the living and the dead into two groups that were then compared statistically. This is meaningless[4], also because we already knew that any differences between the two groups were random. In yet another Christmas issue, the quantum theory arguments were rejected by a physicist [6].

Another trial originally had three authors [7], but the current entry in PubMed lists only two [8], as the senior author subsequently withdrew his authorship. On PubMed, there is reference to an erratum in the journal [9], but our university library has informed us that the page that should describe the withdrawn authorship in the journal does not exist. We have therefore asked the editors of the journal whether the PubMed citation is wrong or whether the erratum was not published in the Journal, in which case the erratum itself is wrong, but have not received any reply despite repeated requests. Similarly, after publication of the trial in 2001, requests for clarifications addressed to the authors and the editors from scientists and journalists were not answered, and not a single critical letter was published in the journal [10,11]. A news release from Columbia University stated that the senior author led the trial, but the vice president noted that the senior author first learned of the trial from the first author six to twelve months after it was completed [10]. One of the two remaining authors was sent to prison [10,11] after 20 years of continuous criminal, fraudulent activities [10,11], and the other remaining author provided incorrect and misleading statements about the research [12,13] after having been challenged by the editor to provide explanations when the scandal broke loose in 2004. The jailed author organised the study, which reported a significantly higher pregnancy rate in the prayer group (50% versus 26%, P = 0.001) after in-vitro fertilisation at a Korean hospital. The prayer was long-distance, as it was carried out in USA, Canada and Australia. All of those who prayed were Christians, as opposed to the Korean patients. Another curiosity is that the Catholic church condemns in-vitro fertilization. It would therefore be equally reasonable to conclude that the responsive god is not very well represented by the Pope, as to conclude that one should pray for those seeking in-vitro fertilisation. The recent statement by the Pope that condoms do not help against the problem with HIV in Africa but that they, on the contrary, increase it [14], is also evidence that a caring, loving god is not well represented by the Pope.

Scientific misconduct seems to have been involved in a third trial [6,15], which was originally included in the Cochrane review but is now excluded, not because of suspected misconduct, but because the intervention was distance healing and not prayer.

### Interpretation of the results

The authors of the Cochrane review are generally cautious but there are notable exceptions. They report a significant effect on death and discuss the huge heterogeneity between the trials, but in violation of their Methods section they did not perform a random effects analysis, which would have shown a non-significant effect.

The authors found one study that reported an *increased *risk of surgical complications due to prayer, but only if the patients were aware that people prayed for them. Instead of discussing the plausibility of this finding, or considering that knowledge of the intervention did not affect the other outcomes in this or other included trials, the authors concluded that people intervening with prayer should be "cautious about informing the recipient" when it comes to surgery and that managers and policymakers may wish to exercise some caution about "praying at the bedside of those who are about to have a surgical operation".

When discussing the effect of prayer on the "clinical state", the authors argue that the lack of effect might be because the participants only received prayer for 14 days, and do not consider the far more plausible explanation that the observed lack of effect is because there is no effect.

The theological reasoning leads to a tautology: "A caring God may not wish to prolong suffering, so death therefore might be a positive outcome of prayer". This is a perfect immunization of the hypothesis that makes trials of prayer meaningless. If people survive, it is good for them, and if they die, it is also good for them. The reasoning is based on the assumption of an omnipotent and all-knowing god. But if that were true, why should we then try to influence our fate when such a god already knows what is best for us?

To their credit, the authors mention their Muslim and Christian backgrounds as potential conflicts of interest. That is probably why they consistently speak about god in singular, although some religions have many gods and spirits, as it was also the case in the Middle East about 2,000 years ago [16].

We have informed the editor of the Cochrane review about the major problems. He suggested we published a comment alongside the review, which we have done. He also assured us that the review was not a joke, which we had hoped it was.

## Conclusion

The Cochrane review's mixture of theological and scientific arguments is unsound and unhelpful and would, if accepted, make all scientific endeavours meaningless. The review fails badly to live up to the high standards required for Cochrane reviews and we therefore suggest it be withdrawn.

## Competing interests

We all work at The Nordic Cochrane Centre and have published Cochrane reviews.

## Authors' contributions

We are all doctors; AH also has a degree in philosophy, and PCG is also a biologist, with bugs as his specialty. KJJ wrote the first draft, PCG wrote the following ones and searched the literature. The idea of writing this paper started when PCG wrote a chapter on alternative medicine to a textbook of internal medicine and via healing stumbled across the Cochrane review on prayer that he first thought was meant to be a joke. All authors contributed to manuscript revisions.
